# #WhyIDoIt: A Multidisciplinary Wellness Initiative in an Academic Emergency Department

**DOI:** 10.5811/westjem.2022.4.55813

**Published:** 2022-08-10

**Authors:** Nancy Jacobson, Riley Westein, Rachel Nordstrom, Alicia Pilarski

**Affiliations:** Medical College of Wisconsin, Department of Emergency Medicine, Milwaukee, Wisconsin

## Abstract

**Introduction:**

Healthcare clinicians in critical care settings such as the emergency department (ED) experience workplace stressors and are at high risk for burnout. This correlates with substance abuse, suicidality, career dissatisfaction, early retirement, and suboptimal patient care. Therefore, recognizing, and mitigating, burnout is critical to a healthcare worker’s health and wellbeing. While gratitude and positive psychology are shown to increase resilience and decrease burnout, no prior studies have examined specific ED care team motivators for continued career satisfaction and workplace engagement. To increase the wellness in our ED, we implemented a wellness initiative titled #WhyIDoIt. Our goal was to have all care team members share what motivates them to work in our ED.

**Methods:**

Participants were asked what motivates them in the workplace. We gathered responses each February for three consecutive years, 2017–2019, at our academic Level I trauma center. Emergency department clinicians, nurses, and staff were recruited to participate at grand rounds, nursing huddles, and sign out. Participants self-selected to contribute by writing their response on a sticky note and posting it in the department. After three years of implementing this initiative, we analyzed the collected qualitative data using thematic analysis based on grounded theory. Submissions were subjectively categorized into initial themes and then reconciled into three overarching classifications.

**Results:**

In total, we collected 149 responses. Themes included team work (35, 23.5%), pride in a unique skill set (26, 17.4%), helping patients in a time of need (26, 17.4%), teaching/learning opportunities (15,10.1%), humor and levity (14, 9.4%), building relationships with patients (11,7.4%), financial motivation (9, 6.0%), patient gratitude (7, 4.7%), and philosophical and moral motivators (6, 4.0%). These themes were reconciled into three overarching classifications including team-centered motivators (76, 51%), patient-centered motivators (37, 24.8%), and reward-centered motivators (36, 24.2%).

**Conclusion:**

Responses that showed the greatest motivator for ED clinicians and nurses were team-centered. This highlights the importance of relationship building and a sense of shared purpose and suggests that future workplace well-being initiatives should include strengthening and maintaining professional team relationships.

## INTRODUCTION

Burnout is defined as a psychological syndrome with emotional exhaustion, depersonalization, and reduced personal accomplishment.^1^ Less than 10 years after its initial definition, this concept was applied to the healthcare field. Emergency physicians (EP) are consistently exemplars of one of the most burned-out specialties.^2^ Additionally, 29–44% of emergency department (ED) nurses experience burnout.^3^,^4^ A growing body of literature identifies contributors to EP burnout. Shift work results in the loss of sleep and circadian rhythm. This has correlated to diminished personal wellbeing, decreased job satisfaction, and pitfalls in patient care. Work-related factors include high intensity work, task switching, uncertainty, second victim syndrome, and patient and colleague disrespect.^5^ Emergency physicians fear litigation and face more malpractice claims than the average physician.^6^ While no specific study focuses on the prevalence or sources of burnout in ED care team members such as advanced practice providers, pharmacists, technicians, social workers, unit coordinators, or environmental services employees, these team members are exposed to many of the same burnout risks as EPs and ED nurses.

Burnout results in significant consequences. It correlates with an increase in medical errors, lower quality patient care, and decreased patient satisfaction.^7^ Emergency physicians experiencing burnout are more likely to reduce their work hours, pursue administrative positions, or leave healthcare completely.^8^,^9^ Rates of depression for EPs vary from 12.1–19.3%. This leads to increased substance abuse and suicidal ideation. It is estimated that 300–400 physicians die by suicide each year.^2^,^5^ While EPs clearly experience the consequences of burnout, other care team members experience consequences as well. Burnout in nurses correlates with ineffectiveness, depression, apathy, absenteeism, and attrition. As it does in physicians, burnout in nurses negatively impacts patient safety and patient satisfaction. Specific data on the consequences of burnout for other ED care team members does not exist; however, similar stressors are present throughout an entire healthcare team.

Due to the negative impacts of burnout on healthcare professionals, much effort has been dedicated to burnout mitigation. Prior research in this field has identified gratitude, team-based support, and resiliency as successful burnout mitigation techniques.^10^,^11^ Gratitude exercises, thankful events, and other motivational programs have been shown to increase enthusiasm and interest in patient care and alleviate depression.^10^ Teamwork initiatives consistently improve physician burnout and increase physician job satisfaction. Team-based efforts additionally minimize stress, improve peer-to-peer communication, and create a culture of appreciation, support, and engagement.^11^ Resiliency is the ability to adapt, rebound, and overcome adversity, and is a proven burnout mitigator. Mindfulness, gratitude, and positive psychology have been shown to increase resiliency and decrease burnout.

To date, research has not specifically aimed to identify protective motivating factors in the workplace for ED care team members. Our aim was to increase the wellbeing of our ED care team members and identify common workplace motivators. To achieve these objectives, we implemented a wellness initiative titled #WhyIDoIt. Our goal was to invite all care team members to share each day what inspires and motivates them to work in the challenging clinical environment of the ED.

**Population Health Research Capsule**

What do we already know about this issue?

*Emergency department healthcare team members experience high rates of burnout, with downstream effects. Dedicated efforts have been made toward burnout mitigation*.

What was the research question?

*Identify workplace motivators of an ED healthcare team in a challenging clinical environment*.

What was the major finding of the study?

*The most frequent workplace motivator was team-centered (51%), followed by patient- and reward-centered motivators (25% each)*.

How does this improve population health?

*Future efforts to improve healthcare team wellness should focus on proven workplace motivators. In the ED, team-based motivation is prevalent*.

## METHODS

This was an observational study of a convenience sample of ED care team members, which took place during the month of February for three consecutive years, 2017–2019, at a Level I academic trauma center ED in a mid-sized midwestern city. All ED care team members were invited to participate regardless of team role, scope of practice, level of training, clinical experience, or full-time employment. Participants were asked what motivates them in the workplace. We recruited ED healthcare professionals (physicians, residents, nurse practitioners, and physician assistants) and staff (nurses, emergency technicians, social workers, health unit coordinators, pharmacists, security, environmental services, transportation, registration) to participate via e-mail and in person at grand rounds, nursing huddles, and sign out. Clinicians and staff were invited to participate as many times as they wished. Participants self-selected to contribute by writing their response(s) on a sticky note and posting it on a board in the department. Responses were either anonymous or identified at the submitter’s discretion.

After three years of implementing this initiative, we analyzed the collected qualitative responses using thematic analysis. Most were submitted anonymously, without identifying information. For submission in which the participant included their name or role, this portion of the submission was disregarded during coding. Submissions were then analyzed and categorized into clusters of meaning by open coding. We assembled these clusters of meaning into initial themes and then reconciled them into three overarching classifications. The first year of submissions was coded in completion prior to coding the subsequent two years’ submissions. This allowed the addition of submissions until theoretical saturation was achieved. Coding was conducted by the authors of this study.

## RESULTS

In total, we collected 149 responses. Themes included teamwork (n = 35, 23.5%); pride in a unique skill set (n = 26, 17.4%); helping patients in a time of need (n = 26, 17.4%); teaching/learning opportunities (n = 15, 10.1%); humor and levity (n =14, 9.4%); building relationships with patients (n = 11, 7.4%); financial motivation (n = 9, 6.0%); patient gratitude (n = 7, 4.7%); and philosophical and moral motivators (n = 6, 4.0%). These themes were reconciled into three overarching classifications including “team-centered motivators” (n = 76, 51%), “patient-centered motivators” (n = 37, 24.8%), and “reward-centered motivators” (n = 36, 24.2%). The figure illustrates the distribution of these themes.

## DISCUSSION

The most frequently referenced workplace motivators for ED care team members in the #WhyIDoIt initiative were team-centered. This confirms a growing body of literature demonstrating a strong correlation between both physician and nursing well-being and colleague support.

*“I love the people and I am so blessed to work with brilliant minds both at the faculty level and staff level. It’s like a second family, and if I have to be here, it should be a warming and welcoming place and it most certainly is!”*

*“Because I love being part of such a great team!”*

*“Together we are amazing.”*

*“I get to work with some of the most amazing, supportive, and caring ED Staff.”*

*“Working with residents and realizing that the future of EM is bright. “*

Colleague support not only improves well-being independently but can also mitigate burnout. Social support of colleagues is positively correlated with job satisfaction, well-being, and reducing job stress.^11^ Additionally, social belonging was associated with well-being among surgical residents, and engagement in students in the STEM fields (medicine and all the biological sciences as well as mathematics, engineering, and computer science).^12^ Nursing well-being and compassion satisfaction are correlated with manager support.^13^ Nonetheless, clinical care in the modern era is increasingly isolating. Physicians spend more time in front of computers, with fewer in-person interactions with colleagues. The results of this study, within the context of the pre-existing literature, would suggest that future workplace initiatives to maintain and improve team relationships would be beneficial.

While the most common reason cited by physicians for choosing a career in medicine was to help patients, this comprised only 17.4% of total submissions in the #WhyIDoIt initiative as part of the patient-centered category.

*“To add comfort to a person’s worst day.”*

*“Because I can make a difference in someone’s life.”*

*“I do this job for the people! Oh, and the great stories!”*

Interestingly, prior research shows the strongest correlator with physician well-being is the physician-patient relationship.^14^ Significant positive and negative correlations are described, suggesting this relationship provides the most gratifying experiences but also causes emotional stress. Emergency physicians often do not have a longitudinal relationship with their patients and are faced with giving bad news or meeting patients under stressful circumstances. Additionally, the patients whom EPs help the most on any given shift are often the sickest and may not remember their ED encounter at all. This unique aspect of EM may impact the extent to which EPs are motivated by building a patient relationship.

The least frequently occurring workplace motivators were reward-centered. Healthcare professionals have previously been cited as contributing to their own lack of well-being by prioritizing work and patients over self-care.^5^

*“When you get a sweet patient who thanks you for taking care of them.”*

*“When you have the patients that truly appreciate everything you do!”*

*“I do it for the little old ladies who hold your hand and say thank you.”*

Interestingly, only 24.2% of submissions mention personal reward as a workplace motivator. Most submissions are patient- or team-centered motivators, meaning most submitters are motivated by interpersonal relationships, or being of benefit to someone else. Perhaps this speaks to the altruism of professionals who pursue a career in healthcare “to help others.” Perhaps the “other” is not always the patient, but a colleague, a care team member, or trainee. This suggests that future efforts may be directed toward developing skillsets that empower care team members to support each other, such as pairing new trainees with mentors, teaching team building as a learned skill, developing peer support, or allowing time for team recognitions at clinician or staff meetings. The results of this study suggest that a care team member may experience motivation not only from receiving support from a peer but also giving support to others.

## LIMITATIONS

Qualitative research is inherently subjective because the data analysis and interpretation is done by the research team. The research team for this study was comprised entirely of emergency medicine (EM)-trained physicians, while the convenience sample of study subjects included all care team members. Further, this initiative was performed at a Level I academic trauma center ED. This clinical practice environment may foster a greater team-centered approach, and some of the responses were related to working with EM residents and medical students. Consequently, generalizability to a non-academic setting may be limited. Additionally, the described wellness initiative was developed through the EM resident wellness committee. This may introduce a proclivity towards physician-centered thinking. However, messaging was sent via email by staff leadership, and participation was encouraged in staff meetings and department-wide huddles. Collaborating with respective staff leaders to message and encourage participation may have increased submissions from a variety of care team members.

Participants self-selected to participate in this wellness initiative and study. One may surmise that care team members who are burned out may be less likely to participate in workplace initiatives. Therefore, results may disproportionately represent the less burned-out members of the ED care team. This was mitigated by including #WhyIDoIt at physician, nursing, and other staff sign-outs. This may have prompted individuals to submit responses who were not otherwise internally motivated to participate.

## CONCLUSION

Responses show the most frequent workplace motivators for ED care team members are team-centered. Meanwhile, reward-centered motivators were the least frequently mentioned. This highlights the importance of relationship building and a sense of shared purpose and suggests that future workplace well-being initiatives should include strengthening and maintaining professional team relationships.

## Figures and Tables

**Figure:**
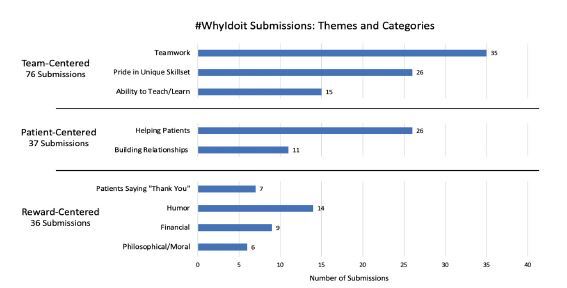
Total number of #WhyIdoit submissions per theme for team-centered, patient-centered, and reward-centered categories.
